# A simple and effective evidence-based approach to asthma management: ICS-formoterol reliever therapy

**DOI:** 10.3399/bjgp24X736353

**Published:** 2024-01-26

**Authors:** Mark L Levy, Richard Beasley, Bev Bostock, Toby GD Capstick, Michael G Crooks, Louise Fleming, Daryl Freeman, Viv Marsh, Hitasha Rupani, Andy Whittamore, Peter J Barnes, Andrew Bush

**Affiliations:** Kenton Bridge Medical Centre, London, UK.; Medical Research Institute of New Zealand, Wellington, New Zealand; School of Medicine, Southampton University, Southampton, UK.; Association of Respiratory Nurses, UK; Mann Cottage Surgery, Moreton-in-Marsh, UK.; Consultant pharmacist, Pharmacy Department, St James’s University Hospital, Leeds, UK.; Hull York Medical School, University of Hull, Hull, UK; Hull University Teaching Hospitals NHS Trust, Hull, UK.; Imperial College Healthcare Trust, London, UK.; Norfolk Community Health & Care, Norwich, UK; Norfolk & Waveney Integrated Care Board, Norwich, UK.; Clinical lead for children and young people’s asthma transformation; Black Country Integrated Care Board, Wolverhampton, UK.; School of Medicine, Southampton University, Southampton, UK; University Hospital Southampton NHS Foundation Trust, Southampton, UK.; Asthma + Lung UK, London, UK.; Airway Disease Section, National Heart & Lung Institute, London, UK.; National Heart and Lung Institute, UK; Imperial Centre for Paediatrics and Child Health, Imperial College London, London, UK; Royal Brompton & Harefield NHS Foundation Trust, London, UK.

## Introduction

**Figure 1.**
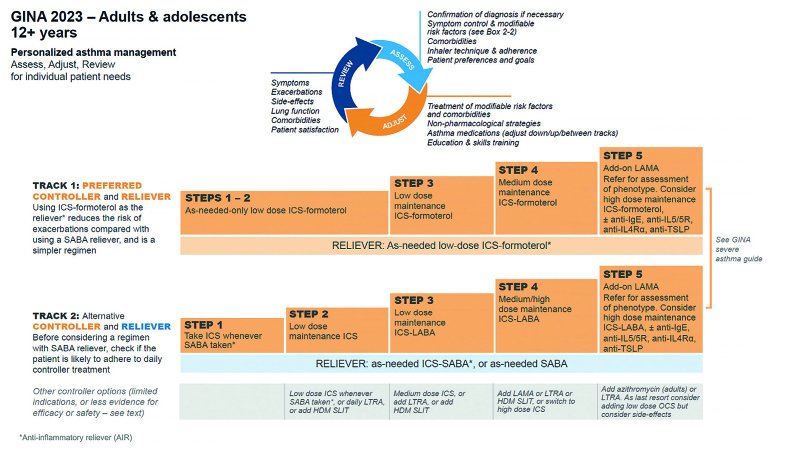
GINA treatment figure for adults and adolescents ≥12 years. The GINA track 1 (preferred) and track 2 (alternative) approaches for adults and adolescents ≥12 years. Before starting, stepping up or down, or switching between tracks, patients should be assessed using the ‘assess, adjust, review’ cycle shown at the top of the figure. GINA = Global Initiative for Asthma. HDM = house dust mite. ICS = inhaled corticosteroid. LABA = long-acting β_2_-agonist. LAMA = long-acting muscarinic antagonist. LTRA = leukotriene receptor antagonist. OCS = oral corticosteroids. SABA = short-acting β_2_-agonist. SLIT = sublingual immunotherapy. Source: GINA.^5^ Reproduced with permission.

Resistance to changing clinical practice in response to scientific evidence is common. Complacency with asthma, and the entrenched overprescribing and overreliance on short-acting β_2_-agonist bronchodilators (SABAs) in asthma, persists in many parts of the world, including in the UK.^1^^–^^4^ Despite the known morbidity and mortality risks associated with regular and/or excessive use of SABAs, most countries have failed to implement the alternative evidence-based, anti-inflammatory reliever treatment (AIR), as recommended by the Global Initiative for Asthma (GINA) as a better and simpler way to manage asthma (Figure 1).^5^ This commentary, using the UK as an exemplar, aims to galvanise these countries to adopt an evidence-based and more effective approach to the management of asthma to reduce the risk of severe attacks.

## UK has among the worst asthma outcomes of high-income countries worldwide

It has been 10 years since the UK National Review of Asthma Deaths^1^ highlighted major preventable factors related to asthma attacks and deaths, which included overuse of SABAs, underprescribing of inhaled corticosteroids (ICS), and failure of healthcare professionals to take asthma seriously as a chronic disease that may kill patients.^3^^,^^4^ The Asthma + Lung UK Annual Survey concluded that only a third of people with asthma received even the most basic level of care in 2022.^6^ Limited access to busy GPs and specialist respiratory nurses, leading to management by staff with inadequate respiratory training or skills, low staffing levels and funding for adult and paediatric consultant respiratory physicians, as well as low referral rates to specialists for people with severe asthma,^2^^,^^7^ are indications that asthma is just not taken seriously enough in the UK.

The UK asthma outcomes are among the worst in Europe, with higher rates of preventable attacks, unscheduled care visits, hospital admissions, deaths, as well as higher medical costs.^1^^,^^8^^,^^9^ In addition to the personal, societal, and healthcare burden of asthma attacks, they contribute to the potentially serious adverse effects of repeated courses of oral corticosteroids.^10^ Traditionally, SABA inhalers are prescribed as the first step in managing asthma and as a result many people thought to have mild asthma are treated with SABA alone.^1^ However, people with supposedly mild asthma have asthma attacks and asthma-related deaths.^1^ From school age and above, asthma of any severity is almost invariably an eosinophilic airway disease and therefore requires ICS-based therapy. SABA monotherapy, a treatment strategy that fails to alleviate inflammation and may even worsen it, is therefore illogical.

NHS England’s Investment and Impact Fund (IIF) 2022/2023 provided a potential solution, with primary care providers incentivised to reduce SABA over-use and increase ICS prescribing in asthma. However, the IIF scheme was amended and currently focuses primarily on healthcare access, thereby missing an opportunity for a step change in asthma care. The presence of two infrequently updated and contradictory UK asthma guidelines is also not helpful.^12^

Evidence of the harmful effects of SABA use, including risk of death, has been available for over six decades, especially when used without ICS.^13^^,^^14^ During this period, extensive evidence of the efficacy of ICS has accumulated, initially when taken as regular scheduled maintenance treatment, and more recently as a component of a reliever inhaler.^15^^–^^18^ As a result, GINA made groundbreaking recommendations in 2019 for the use of ICS-formoterol ‘as needed’ for symptoms, either alone or as maintenance and reliever therapy in adolescents and adults with asthma (see Figure 1, track 1).^5^ In adults and adolescents there is compelling evidence that this approach reduces the risk of severe asthma attacks when compared with the use of a SABA reliever, across the spectrum of asthma severity.^15^^–^^18^

## Anti-inflammatory reliever (AIR) therapy: evidence for an alternative approach

Adherence by asthma patients to regularly scheduled ICS-based maintenance treatment is poor, and many rely on SABAs as their mainstay of treatment, which is understandable because of the immediate relief of symptoms that is obtained with their use.^4^ An obvious solution is to also deliver the ICS preventer drug treatment via the same delivery system as a fast-onset reliever, in a so-called ‘2 in 1’ inhaler. This form of treatment is called anti-inflammatory reliever (AIR), where an ICS is combined with either a SABA or formoterol, a fast-onset, long-acting, β_2_-agonist [LABA], and used as a reliever treatment. In this way the ICS dose is titrated according to changes in symptom frequency and/or severity through the vehicle of bronchodilator reliever use and avoids SABA monotherapy in patients who are poorly or non-adherent to their maintenance ICS-based therapy. In terms of ICS-SABA reliever therapy, combination products are only available in a small number of countries and are not available in the UK or Europe.

There are two forms of AIR therapy with ICS-formoterol: first, maintenance and reliever therapy (called MART) for use in moderate and severe asthma (GINA steps 3, 4, and 5) and, second, ICS-formoterol reliever alone without maintenance ICS treatment in mild asthma (GINA steps 1 and 2) (Figure 1). There is no current evidence for ICS-formoterol reliever therapy in patients taking other maintenance (non-formoterol) combination ICS/LABA treatments with LABAs such as salmeterol and vilanterol. The evidence for ICS-formoterol MART is based on budesonide-formoterol and beclomethasone-formoterol, with these products licensed worldwide for many years. The evidence for ICS-formoterol reliever alone is solely based on budesonide-formoterol, which is now licensed in 48 countries, including in the UK. The AIR regimen is simple to use with patients only needing to use a single inhaler device even if they have severe disease and are using MART therapy, and, for patients with mild disease, as-needed AIR therapy alone avoids the need to take ICS medication every day, a regimen to which many patients do not adhere.

MART reduces severe exacerbations compared with ICS or ICS-LABA plus SABA reliever, with similar symptom control.^15^^,^^17^ For example, in a systematic review and meta-analysis of randomised clinical trials (*N* = 22 748 patients),^17^ ICS-formoterol MART regimens reduced the risk of severe exacerbations compared with the same-dose maintenance ICS-LABA and SABA reliever by 32% (risk ratio [RR] 0.68 [0.58–0.80]). Importantly, ICS-formoterol MART reduced the severe exacerbation risk by 23% (RR 0.77 [0.60–0.98]) compared with doubling the maintenance dose of ICS in ICS/LABA maintenance and SABA reliever.

ICS-formoterol reliever alone reduces severe exacerbations compared with SABA monotherapy in mild asthma.^15^^,^^16^ In a Cochrane systematic review of studies involving 9565 patients,^16^ as-needed ICS-formoterol compared with as-needed SABA reduced severe exacerbations by 55% (odds ratio [OR] 0.45 [0.34–0.60]) and an asthma-related hospital admission or emergency department or urgent care visit by 65% (OR 0.35 [0.20–0.60]). Furthermore, ICS-formoterol reliever alone in mild asthma compared with maintenance ICS plus SABA in two separate inhalers reduced severe exacerbations by 21% (OR 0.79 [0.59–1.07]) and an asthma-related hospital admission or emergency department or urgent care visits by 37% (OR 0.63 [0.44–0.91]). This greater efficacy was achieved despite patients inhaling less than half the mean daily ICS dose than those on regular ICS therapy, suggesting that titrating the dose of ICS, when administered through the vehicle of bronchodilator use, is more important in determining efficacy than the total ICS dose. It is likely that the use of LABA rather than SABA reliever contributed to improved outcomes. In contrast with the findings relating to severe exacerbations, there are no clinically important differences in lung function or asthma symptom control between the ICS-formoterol reliever and maintenance ICS plus SABA reliever regimens.^16^

There is a shocking lack of evidence in children aged 4–11 years, in which group there is only one small study showing efficacy of an ultra-low ICS/formoterol MART regimen.^19^ The UK Health Technology Assessment Programme (HTA) has funded a trial to address this, and it is essential that as many children as possible are recruited to fill this evidence gap. There are also ongoing studies in New Zealand and South Africa to address this evidence gap.

The lack of clear national guidance means that there has been very patchy implementation of these AIR treatment strategies in the UK. One local UK initiative has piloted an intervention to support implementation of guideline-recommended care, resulting in a SABA-free MART strategy in 45% of 864 reviewed asthma patients.^20^ This led to reduced SABA prescribing, increased ICS uptake, and fewer asthma attacks. The feasibility of MART implementation was confirmed with over 70% of patients still using MART after 1 year and ∼85% doing so completely SABA-free. Similar projects rolling out in other centres report similar uptake of SABA-free MART, for example, with 66% of 472 reviewed asthma patients in one Primary Care Network accepting the change in regimen, which may reflect increased confidence in implementing this treatment strategy (T Capstick, personal communication).

The authors are aware that some Integrated Care Systems have already or are in the process of adopting ICS-formoterol AIR treatment regimens. While these examples demonstrate the ability for change to take place at a local level, the lack of national direction has the potential to drive greater variation in care and contribute to health inequalities.

## Call to action

**Figure 2.**
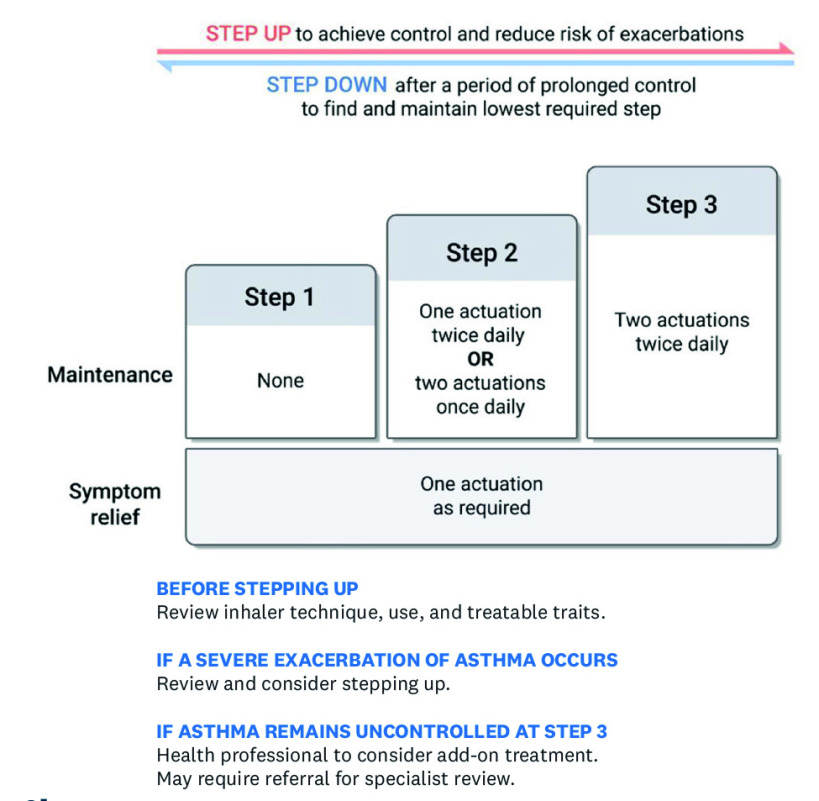
Example of a three-step AIR asthma management guideline, based on budesonide-formoterol 200 ug/6ug, the only ICS-formoterol product with evidence of efficacy/safety across the range of asthma severity. In practice this approach can be implemented through use of an AIR action plan, for example, at: https://bit.ly/41qBooo. Asthma + Lung UK has a MART action plan available in nine different languages at: https://bit.ly/3R8TCGj, with patient information at: https://bit.ly/3sXiqJt and guidance for clinicians at: https://bit.ly/3RsSj6y. An ICS-formoterol AIR-only action plan is due for release by Asthma + Lung UK in early 2024. Source: Asthma + Respiratory Foundation NZ.^11^ Reproduced with permission.

We now propose immediate implementation of strategies, in the UK and elsewhere, to replace the use of SABA inhalers for relief of symptoms in those over the age of 12 years with ICS-formoterol AIR therapy. One opportunity is to implement the New Zealand strategy of a simple three-step asthma guideline (Figure 2)^11^ consistent with the recommended GINA-preferred treatment track utilising AIR therapy,^5^ along with an associated asthma action plan.^11^ We suggest that for adolescents and adults:

- ICS-formoterol reliever alone for all newly diagnosed patients with mild asthma, escalating to MART if still uncontrolled. If this does not achieve control, referral to an asthma specialist (GP, asthma-trained nurse, or respiratory consultant) is mandated;
- immediate transfer of those aged 12 years and over using SABA alone (including those who only use their SABA occasionally) to ICS-formoterol reliever alone, and anyone using ICS plus SABA for relief, to an ICS-formoterol MART regimen;
- changing those treated with maintenance ICS-LABA plus SABA for relief to ICS-formoterol MART therapy; and
- patients taking medium- or high-dose ICS/LABA plus a SABA who have poor asthma control or a recent exacerbation should be switched to medium-dose ICS-formoterol MART. All those whose asthma remains poorly controlled despite such optimisation of treatment should be referred for specialist assessment of severity, inflammatory biomarkers, phenotyping, and possible biological treatment.^5^

While there may be situations where continuation of SABA reliever alongside ICS containing preventive treatment are appropriate, such as for adolescents and adults on maintenance ICS or ICS/LABA plus SABA reliever regimens with good asthma control and at low risk of attacks, high adherence, and good inhaler technique, or children under the age of 12 years where evidence for AIR is still needed, we propose that, if we are to achieve a meaningful improvement in asthma outcomes in the UK and other countries in which availability is not restricted, SABA-containing regimens should be the exception rather than the norm. However, the AIR regimen should not be the single focus implemented in isolation, but should be part of a comprehensive approach in which the diagnosis of asthma is confirmed, and overlapping disorders, comorbidities, and environmental and lifestyle factors are considered, including patient education and teaching of inhaler technique.^14^

Clearly, *there is a need for a change in the way asthma is managed in the UK*. Leadership to facilitate and motivate change and patient education is needed to increase awareness of the risks of SABA overuse and the benefits of AIR therapy in asthma. A new national asthma strategy, incorporating both AIR therapy alone and MART, has a number of potential advantages including reduced risk of severe asthma exacerbations, reduced healthcare costs, and reduction of unscheduled care with reduced workload for GPs and hospitals. Above all, it will improve the current scandalously poor UK asthma outcomes.
